# Ornithine transcarbamylase deficiency: A diagnostic odyssey

**DOI:** 10.1002/jimd.12530

**Published:** 2022-07-10

**Authors:** Ina Knerr, David Cassiman

**Affiliations:** ^1^ National Centre for Inherited Metabolic Disorders Children's Health Ireland (CHI) at Temple Street Dublin Republic of Ireland; ^2^ University College Dublin (UCD) UCD School of Medicine Dublin Republic of Ireland; ^3^ Department of Gastroenterology‐Hepatology and Metabolic Center University Hospital Leuven Leuven Belgium

In two separate publications in *Journal of Inherited Metabolic Disease* (2022), Hertzog^1^ and co‐authors in Australia and Han/Bjornsson et al.^2^ from the U.S. and Iceland have further elucidated the molecular pathophysiology in a subgroup of predominantly male patients with late‐onset ornithine transcarbamylase deficiency (OTCD), an X‐linked urea cycle disorder. The authors put the spotlight on pathogenic variants affecting the regulatory regions of the *OTC* gene, specifically the *OTC* promoter variant c.‐106C>A. This leads to impaired promoter function of the *OTC* gene and may present as late‐onset OTCD in apparently healthy individuals; however, when metabolic stress is increased, impaired upregulation of *OTC* gene activity can then be followed by hyperammonaemia.

Parallel to both of these reports, two additional males (16 and 20 years of age, respectively) with, thus far, clinically very mild late‐onset OTCD, were diagnosed in Ireland by our group. They had been under surveillance with “sick day” management and L‐arginine supplements due to a positive family history of hyperammmonaemia with a biochemical diagnosis of OTCD in their older brother. This Irish family had extensive genetic testing done, including *OTC* gene sequencing in different diagnostic laboratories, which came back negative. The two younger brothers' diagnosis was based on an allopurinol load performed 10 years previously. Further to diagnostic findings of Jang et al.^3^ which describe the pathogenic variant c.‐106C>A in the promoter region of the *OTC* gene, a diagnosis of OTCD could be genetically confirmed for the two Irish patients. Whole exome sequencing also verified that no other pathogenic variant was identifiable relating to their biochemical findings. These biochemical results include, for example, normal plasma ammonia measurements to date and, intermittently, marginally raised transaminases.

The difficult diagnostic journey with its serendipitous outcome, described by Hertzog and collaborators, underscores how metabolic medicine is sometimes not unlike detective work, particularly when confirmatory testing comes back negative, but clinical concerns remain. In the three cases presented by Hertzog et al., routine Sanger sequencing of the *OTC* gene did not identify a disease‐causing variant, nor did next generation sequencing/urea cycle disorder gene panel. Along these lines, both publications send out a general warning in these sequencing‐oriented times: a clinical or biochemical suspicion should not be ruled out, based only on negative DNA sequencing results.

The difficulties which may arise are wide‐ranging, including the risk of either a near‐missed diagnosis or an incomplete diagnosis with no confirmation. If the familial pathogenic variant is not known (and there may be many more as yet undiscovered pathogenic variants in gene regulatory regions) and biochemical testing comes back nondiagnostic, we may still not be able to rule out OTCD, for example, in relatives at risk. Where the diagnosis of OTCD cannot be established but poses an active concern, for example, in family members at risk, preventive measures, and surveillance, together with appropriate counseling with active patient/parent involvement may be reasonable approaches until the evidence becomes clearer.

In the study conducted by Han and Bjornsson and collaborators, published in the *Journal of Inherited Metabolic Disease* (2022), the diagnostic profile of the proband's brother was also included. This boy had been reported to have died by drowning in his teenage years, 20 years prior to the presentation of his adult brother. Taking a full family history and considering the medical examiner's findings were key, the latter showing unusual findings, such as cerebral edema, which are not typical for acute drowning but consistent with hyperammonaemia, along with reported confusion and vomiting that preceded this boy's fall and drowning. The medical examiner's findings became crucial in later years, as they provided a context for understanding the presentation of the other brother, who had also developed confusion and cerebral edema due to hyperammonaemia and was subsequently diagnosed with OTCD.

Han/Bjornsson and collaborators furthermore present a large Caucasian family tree with a number of males affected by the *OTC* gene c.‐106C>A variant. Their symptoms were wide‐ranging, with entirely asymptomatic males with normal biochemistry at one extreme to overwhelming hyperammonaemic encephalopathy with possible death at the other. Invasive diagnostic techniques, such as OTC enzyme activity in liver biopsy, have become less and less available due to the availability of new technologies as DNA sequencing. This has directly contributed to the complexity of diagnosing challenging cases. We have to stay mindful of the pitfalls of sequencing methods, and we should strive to maintain parallel functional/biochemical diagnostic technologies, for example, for confirmation of genetic variants of unknown significance and for false‐negative sequencing cases.

The *OTC* c.‐106C>A pathogenic variant seems to be extremely rare or under‐reported; however, it appears to be a recurrent variant in males with late‐onset OTCD. There is now growing evidence that it impairs promoter activity, particularly maximal enzyme activity levels under high metabolic stress, possibly by affecting the binding of gene transcription factors. In the absence of metabolic stress and diagnostic biochemical markers, the variant may be present, but, without additional work‐up, can go undetected.

With this in mind, we can conclude that in individuals at risk in whom thorough metabolic work‐up is unrevealing, targeted biochemical surveillance combined with more functional diagnostic technology is necessary. This would include point‐of‐care testing, for example, during episodes of metabolic stress together with the diagnostic utilization of newer molecular genetic technologies and other complementary diagnostic tools. All of this requires active patient engagement with a clear understanding of the situation, including the potential limitations of the diagnostic tests applied. With a spotlight on individuals with suspected inborn metabolic disorder, based on hitherto unrecognized symptoms or extended family history, further pathogenic variants in the DNA regulatory elements will emerge.

## References

[1] Hertzog A , Selvanathan A , Halligan R , et al. A serendipitous journey to a promoter variant: the c.‐106C>A variant and its role in late‐onset ornithine transcarbamylase deficiency. J Inherit Metab Dis Rep, in press. 2022. 10.1002/jmd2.12289 PMC9259394 35822098

[2] Han S , Anderson K , Bjornsson H , Longo N , Valle D . A promoter variant in the OTC gene associated with late and variable age of onset hyperammonemia. J Inherit Metab Dis, in press. 2022. 10.1002/jimd.12524 PMC10323875 35605046

[3] Jang YJ , LaBella AL , Feeney TP , et al. Disease‐causing mutations in the promoter and enhancer of the ornithine transcarbamylase gene. Hum Mutat. 2018;39(4):527‐536. 29282796 10.1002/humu.23394 PMC7388160

